# Generation of Reactive Oxygen Species by Polyenylpyrroles Derivatives Causes DNA Damage Leading to G2/M Arrest and Apoptosis in Human Oral Squamous Cell Carcinoma Cells

**DOI:** 10.1371/journal.pone.0067603

**Published:** 2013-06-28

**Authors:** Kuo-Feng Hua, Pei-Chun Liao, Zhanxiong Fang, Feng-Ling Yang, Yu-Liang Yang, Yi-Lin Chen, Yi-Chich Chiu, May-Lan Liu, Yulin Lam, Shih-Hsiung Wu

**Affiliations:** 1 Department of Biotechnology and Animal Science, National Ilan University, Ilan, Taiwan; 2 Department of Chemistry, National University of Singapore, Singapore, Singapore; 3 Institute of Biological Chemistry, Academia Sinica, Taipei, Taiwan; 4 Agricultural Biotechnology Research Center, Academia Sinica, Taipei, Taiwan; 5 Department of Biomechatronic Engineering, National Ilan University, Ilan, Taiwan; 6 Department of Nutritional Science, Toko University, Chiayi, Taiwan; Roswell Park Cancer Institute, United States of America

## Abstract

Oral squamous cell carcinoma (OSCC) accounts for 5.8% of all malignancies in Taiwan and the incidence of OSCC is on the rise. OSCC is also a common malignancy worldwide and the five-year survival rate remains poor. Therefore, new and effective treatments are needed to control OSCC. In the present study we have investigated the efficacy and associated mechanisms of polyenylpyrroles and their analogs in both *in vitro* cell culture and *in vivo* nude mice xenografts. Auxarconjugatin B (compound **1a)** resulted in cell cycle arrest in the G2/M phase and caspase-dependent apoptosis in OEC-M1 and HSC-3 cells by activating DNA damage and mitochondria dysfunction through the loss of mitochondrial membrane potential, release of cytochrome c, increase in B-cell lymphoma-2-associated X protein level, and decrease in B-cell lymphoma-2 level. Compound **1a**-induced generation of intracellular reactive oxygen species through cytochrome P450 1A1 was identified as a major mechanism of its effect for DNA damage, mitochondria dysfunction and apoptosis, which was reversed by antioxidant N-acetylcysteine as well as cytochrome P450 1A1 inhibitor and specific siRNA. Furthermore, compound **1a**-treated nude mice showed a reduction in the OEC-M1 xenograft tumor growth and an increase in the caspase-3 activation in xenograft tissue. These results provide promising insights as to how compound **1a** mediates cytotoxicity and may prove to be a molecular rationale for its translation into a potential therapeutic against OSCC.

## Introduction

According to the latest report from the Department of Health, Executive Yuan, Taiwan, oral cancer affects a significant number of patients in their economically productive age and approximately 2300 men in Taiwan with an average age of 58.3 years succumb to oral cancer every year. Oral cancer is also a common malignancy worldwide and the incidence of oral cancer continues to increase annually [1]. The usual therapy for oral cancer involves one or more of the following modalities: surgery, chemotherapy and radiotherapy. Unfortunately, despite advances in clinical management, the survival rate remains poor [2], [3]. This strongly underlines the importance of discovering and developing new and effective treatments to improve the prognosis of oral cancer patients.

Apoptosis is one of the important mechanisms of anticancer drug-mediated cell death. It is induced by two major pathways: mitochondrial (intrinsic) pathway and death receptor (extrinsic) pathway. Mitochondrial pathway is activated by the release of proapoptotic factors, such as cytochrome c and apoptotic inducing factor, from the mitochondria into the cytosol. The mitochondrial outer membrane permeability is regulated by the Bcl-2 family proteins, which are the central regulator of cytochrome *c* release and caspases activation [4]. After being released from the mitochondria, cytochrome c can bind to dATP and apoptotic protease-activating factor-1 which results in the activation of caspase-9 and caspase-3. Activated caspase-3 cleaves various substrates, including poly (ADP-ribose) polymerase (PARP), a DNA repair enzyme, thus leading to inevitable cell death [5]. Death receptor pathway involves the Fas and other members of the tumor-necrosis factor receptor family that triggers caspase-8 activation [6]. Caspase-8 directly activates caspase-3 and cleaves Bid, which then triggers the mitochondrial pathway [7]. Reactive oxygen species (ROS) generation has usually been observed during the process of apoptosis in cells subjected to anticancer drugs treatment [8]. Increased ROS level might lead to DNA damage and these damaged cells subsequently undergo either cell cycle arrest to facilitate DNA repair, or induce apoptosis to eliminate the excessively damaged cells [9]. DNA damage might activate p53-dependent apoptosis through inhibiting both the G1/S and the G2/mitosis (M) transitions by directly stimulating the expression of p21WAF1/CIP1, an inhibitor of cyclin-dependent kinases (Cdks) [10]. DNA damage might also activate protein kinases ATM and ATR which subsequently triggers the activation of the protein kinases Chk1 and Chk2, which in turn inhibits Cdc2 by inactivating Cdc25, the phosphatase that normally activates Cdc2 [11].

Conjugated polyenes is an interesting class of widely occurring natural products which have been shown to possess excellent biological properties including antitumor activities [12]. However, the typically small quantities that can be obtained from the isolation of natural sources (fungi or bacteria) often limit its applications. To address this limitation as well as to provide access to structurally diverse analogs of these compounds, we have developed a synthetic strategy that allows conjugated polyenes to be synthesized expediently. In our previous study a class of polyenylpyrroles and their analogs were designed from a hit compound identified in a fungus and compound **1g** was identified as a potent anti-cancer agent against human non-small cell lung carcinoma cell lines A549 [13]. In this study, the compounds synthesized were evaluated for their cell cytotoxicity to four human oral squamous cell carcinoma cell lines.

## Materials and Methods

### Cell Lines and Reagents

**Figure 1:**
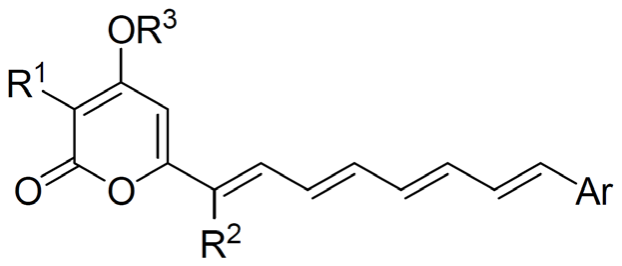
The backbone of the synthesized polyenylpyrroles.

The backbone of the synthesized polyenylpyrroles was shown in Fig. 1 [13]. OEC-M1 and SAS cell lines were provided by Prof. Tzong-Ming Shieh, China Medical University [14], [15]. HSC-3 cell line was obtained from the Japanese Collection of Research Bioresources. SCC-4 cell line was obtained from ATCC (Manassas, VA, USA). OEC-M1 and HSC-3 cells were cultured in RPMI 1640 medium; SAS and SCC-4 cells were cultured in MEM-F12 and DMEM medium respectively. All cells were cultured under standard conditions. 2′,7′-dichlorofluorescein diacetate and 3,3′-diethyloxacarbocyanine iodide (DiOC_2_(3)) were obtained from Molecular Probes (Eugene, OR). Antibodies against caspase-8, PARP, Bcl-2, Bax, Bak, and actin were purchased from Chemicon (Temecula, CA). Antibodies against p-cdc2, cyclin B1, p-chk2, p53, p21, and CYP1A1 were obtained from Santa Cruz Biotechnology Inc (Santa Cruz, CA). Antibodies against caspase-3 and caspase-9 were purchased from R&D Systems (Minneapolis, MN). Antibody against VDAC (anti-porin) was purchased from EMD Millipore (Billerica, MA). All other chemicals were obtained from Sigma Chemical Co. (St. Louis, MO).

### *In vitro* Growth Inhibition Assay

Cells were cultured for 24 and 48 h in 96 wells plate containing media which comprise the tested compound and a final DMSO concentration of 0.1%. Thereafter, 10 µl AlamarBlue® reagent (AbD Serotec, Oxford, U.K.) was added into each well and the cells were further incubated at 37°C for 6 h. The cell viability was determined by the fluorescence intensity detected at 570 nm and 600 nm.

### Cell Cycle Determination and TdT End-labeling Assay

DNA content and cell cycle distribution were analyzed using a Becton Dickinson FACScan Plus flow cytometer. Cytofluorometric analysis was performed using a CellQuestR (Becton Dickinson, San Jose, CA), on a minimum of 10000 cells per sample. Apoptosis was assayed by a TdT-mediated dUTP Nick-End Labeling assay and measured by the FACScan system [13].

### Detection of Mitochondrial Cytochrome C Release

By flow cytometry method: Cells (1×10^5^) were harvested, treated with 100 µl digitonin (50 µg/ml in PBS with 100 mM KCl) for 5 min on ice (until >95% were permeabilized as assessed by trypan blue exclusion), and fixed in 4% paraformaldehyde for 20 min at room temperature. After which, the cells were washed three times in PBS, incubated in blocking buffer (3% BSA, 0.05% saponin in PBS) for 1 h and stained with 1 µg anti-cytochrome *c* antibody (Chemicon, Temecula, CA) overnight at 4°C. Thereafter, the cells were further washed three times and incubated with 1∶200 FITC-labeled secondary antibody for 1 h at room temperature. Cells were analyzed by flow cytometry. By western blotting method: cells with or without treatment were harvested by centrifugation at 600×g for 10 min. After washing once with ice-cold PBS, cell pellets were resuspended in buffer A (20 mM HEPES-KOH, pH 7.5, 10 mM KCl, 1.5 mM MgCl_2_, 1 mM Na-EDTA, 1 mM Na-EGTA, 1 mM dithiothreitol, 0.1 mM phenylmethylsulfonyl fluoride containing 250 mM sucrose). After being chilled on ice for 30 min, the cells were disrupted by 15 strokes of a glass homogenizer. The homogenate was centrifuged twice to remove unbroken cells and nuclei (750×g, 10 min, 4°C). The supernatant was then obtained by centrifugation at 10000×g for 60 min at 4°C. The resulting pellets were identified as the mitochondrial fraction, and supernatants were identified as cytosolic fraction. All steps were performed on ice or 4°C. Cytochrome *c* release into the cytosolic fraction for each condition was assessed by western blotting analysis.

### Measurement of Mitochondrial Membrane Potential (Δψm)

A reduction in mitochondrial membrane potential was monitored by DiOC_2_(3) staining and measured by flow cytometry [13].

### Western Blotting Analysis and Reactive Oxygen Species Detection

Whole cell lysates were prepared from DMSO- or compound **1a**-treated cells and western blotting was performed following earlier described methods [16]. Intracellular H_2_O_2_ was measured by detecting the fluorescence intensity of 2′,7′-dichlorofluorescein, the oxidation product of 2′,7′-dichlorofluorescein diacetate, as described previously [17].

### DNA Damage Assay

Genomic DNA isolation and the apurinic/apyrimidinic (AP) sites of DNA lesions were analyzed as described previously [18]. The number of AP sites was assessed using a DNA damage quantification kit (Abcam, Cambridge, MA) according to the manufacturer’s instructions.

### RNA Interference

OEC-M1 cells (1×10^6^) were transfected with cytochrome P450 1A1 siRNA (sc-41483) or control siRNA (sc-37007) using LyoVec™ transfection agent according to the manufacturer’s protocols (Santa Cruz Biotechnology Inc). After transfection, the cells were incubated for 24 h and then subjected to compound 1a stimulation for further analysis [19].

### Ethics Statement

All animal manipulations were performed in the Laboratory Animal Center of National Ilan University (Ilan, Taiwan) in accordance with the National Ilan University guide for the care and use of laboratory animals. The procedures used were approved by the Animal Care and Use Committee of the National Ilan University. All manipulations were performed under isoflurane anesthesia, and all efforts were made to minimize suffering.

### Antitumor Activity *in vivo*

Female 6–8-week old congenital athymic BALB/c nude (nu/nu) mice were used for the experiment (National Laboratory Animal Center, Taipei, Taiwan). The animals were s.c. implanted with 1×10^7^ OEC-M1 cells into the back of mice. When the tumor reached 80–120 mm^3^ in volume, animals were divided randomly into control and test groups consisting of six mice per group (day 0). Daily s.c. administration of compound **1a**, dissolved in a vehicle of 20% Tween 80 in normal saline (v/v) was performed from days 0 to 4 far from the inoculated tumor sites (>1.5 cm). The control group was treated with vehicle only. The tumor volume was determined by measurement of the length (*L*) and width (*W*) of the tumor three times a week and the mice were weighed at the same time. The tumor volume at day *n* (TV*n*) was calculated as TV (mm^3^) = (*L*×*W* ^2^)/2. The relative tumor volume at day *n* (RTV*n*) versus day 0 was expressed according to the following formula: RTV*n* = TV*n*/TV0. The condition of the mice did not decline significantly during the experiment and no significant signs of suffering were observed. The maximal allowable tumor size is 1000 mm^3^ beyond which action is taken to euthanize the mice. The tumor size of one of the control group mice reached 1012 mm^3^ at day 28, and at the same day all the mice were humanely euthanized by inhalation of an overdose of isoflurane to minimize suffering. Xenograft tumors as well as other vital organs of treated and control mice were harvested for further study.

### Caspase-3 Activity Assay

Tissues lysates were prepared in lysis buffer (0.25 mM sucrose, 1 mM EDTA, 10 mM Tris and a protease inhibitor cocktail (Sigma Chemical Co.). The lysates (50 µg protein) were incubated for 1 h at 37°C in HEPES buffer containing 100 µM concentrations of the specific fluorogenic substrate Ac-DEVD-AMC (*BD Biosciences,* Franklin Lakes, NJ). Caspase-3 activity was measured by monitoring the cleavage of the caspase-3 substrate at excitation wavelength of 360 nm and emission wavelength of 460 nm, respectively using a spectrofluorimeter. Activity was expressed as fluorescence units per milligram of protein per min of incubation.

### Statistical Analysis

All values are given as the mean ± SD. Data analysis was performed by one-way ANOVA with a subsequent Scheffe’ test.

## Results

### Characterization of Compound 1a-induced Apoptotic Cell Death

**Figure 2:**
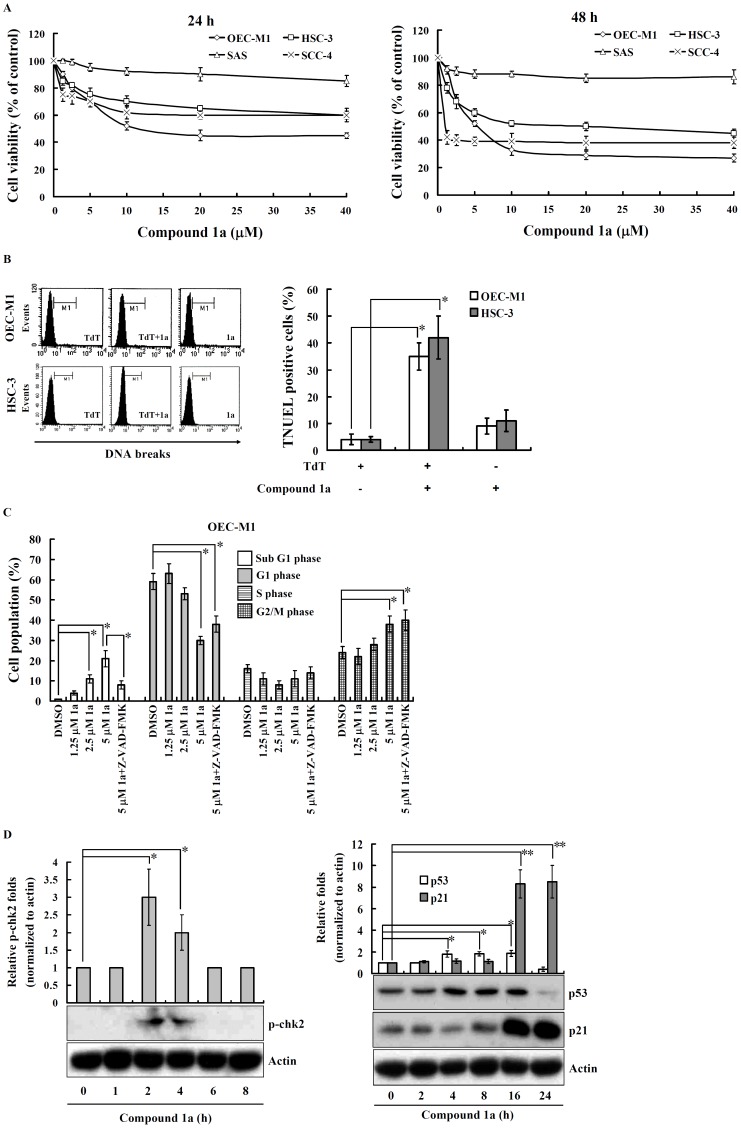
Characterization of compound 1a-induced apoptotic cell death. (**A**) Cells were seeded in 96-well plates, treated with compound **1a** (1.25 ∼ 40 µM) or vehicle (0.1% DMSO) for 24 and 48 h. Cell viability was measured by A*lamarBlue*® assays. The data were expressed as mean ± SD; n = 3. (**B**) OEC-M1 and HSC-3 cells were treated with compound **1a** (5 µM) or vehicle for 24 h, and the DNA breaks were analyzed by flow cytometry based TUNEL assay. The results are representative of those obtained in three different experiments and the histogram shows the quantification expressed as the mean ± SD. *indicates a significant difference at the level of *p*<0.05. (**C**) OEC-M1 cells were treated with various concentrations of compound **1a** as indicated, Z-VAD-FMK (50 µM) plus compound **1a** (5 µM) or vehicle for 24 h. The cell cycle distribution was determined by flow cytometry after PI-staining of the nuclei. The data were expressed as mean ± SD; n = 3. *indicates a significant difference at the level of *p*<0.05. (**D**) OEC-M1 cells were treated with compound **1a** (5 µM) or vehicle for the time as indicated. The expression of p-chk2, p53 and p21 were measured by western blotting. The results are representative of those obtained in three different experiments and the histogram shows the quantification expressed as the mean ± SD. * and **indicate a significant difference at the level of *p*<0.05 and *p*<0.01 respectively.

Compounds **1a**-**n** and **21** at concentration of 20 µM were evaluated for their cytotoxicities against the human oral squamous cell carcinoma cell lines: OEC-M1, HSC-3, SAS, and SCC-4 after 24 h and 48 h treatment with *AlamarBlue*® assay. The anti-cancer drug paclitaxel was used as a positive control for cell death induction (Table 1) [20]. As shown in Table 1, compound **1a** was found to be the most potent compound as it exhibited high cytotoxicity in three cell lines except SAS cells (Cell viability for 48 h compound **1a** treatment: OEC-M1∶29±2%; HSC-3∶50±2%; SCC-4∶38±3%; SAS: 85±3%). To further analyze the cytotoxicity of compound **1a**, OEC-M1, HSC-3, SAS, and SCC-4 cells were incubated with increasing doses of the compound (1.25–40 µM) or DMSO (0.1%, vehicle control) for 24 and 48 h. As shown in Fig. 2A, compound **1a** significantly inhibited the viability of OEC-M1, HSC-3, and SCC-4 cells with IC_50_ of 5 µM, 20 µM, and 1 µM at 48 h, respectively. To gain further insight into the mode of action of compound **1a**, two assays targeting hallmarks of apoptosis, namely the apoptotic DNA fragmentation assayed by terminal deoxynucleotidyl transferase dUTP nick end labeling (TUNEL) assay, and the cell cycle assayed by flow cytometry after propidium iodide (PI)-staining nuclei were performed. As shown in Fig. 2B, results from the TUNEL assay showed that compound **1a** induced 35±9% and 42±11% TUNEL positive cells in OEC-M1 and HSC-3 cells, respectively, indicating that compound **1a** induces apoptotic DNA fragmentation in OEC-M1 and HSC-3 cells. In addition, the effect of compound **1a** on the cell cycle distribution in OEC-M1 cells was determined and the results obtained showed that the cells in G2/M phase increased in a concentration-dependent manner after treatment with compound **1a**, while concomitantly decreasing the percentage of cells in G1 and S phase as compared with the control cells, indicating that compound **1a** induces cell cycle arrest at G2/M phase (Fig. 2C). Moreover, the cells in sub-G1 phase increased in a concentration-dependent manner after treatment with compound **1a**. However these effects were reduced by the pan caspase inhibitor carbobenzoxy-valyl-alanyl-aspartyl-[*O*-methyl]-fluoromethylketone (Z-VAD-FMK), indicating that compound **1a** induced apoptosis through caspase dependent manner (Fig. 2C). Compound **1a** has a similar effect on the cell cycle distribution of HSC-3 cells (data not shown). To investigate the underlying mechanism by which compound **1a** treatment induced cell-cycle arrest at G2/M, we examined the effect of compound **1a** on G2/M phase regulatory proteins, including chk2, p53, and p21. We found that the phosphorylation level of chk2 was augmented after 2–4 h treatment with compound **1a** (Fig. 2D, top panel). The expression levels of p53 starts to increase after 4 h of treatment with compound **1a** and continues increasing till 16 h of treatment. On the other hand, p21 starts to increase at 8 h and peaks at 16 h after treatment with compound **1a** (Fig. 2D, bottom panel).

**Table 1:** Anticancer Activity of Gymnoconjugatin Derivatives against Human Oral Cancer Cells.

					OEC-M1		HSC-3		SAS		SCC-4	
Sample^a^	R^1^	R^2^	R^3^	Ar	24 h	48 h	24 h	48 h	24 h	48 h	24 h	48 h
1a	H	H	Me	3-chloropyrrol-2-yl	45±2^b^	29±2	71±2	50±2	90±2	85±3	60±4	38±3
1b	Me	H	Me	3-chloropyrrol-2-yl	102±1	100±1	98±3	100±2	102±3	100±4	70±2	50±3
1c	*n*Bu	H	Me	3-chloropyrrol-2-yl	80±3	82±3	83±3	70±3	90±3	9 0±2	72±3	55±2
1d	H	Me	Me	3-chloropyrrol-2-yl	85±3	85±2	90±2	82±2	90±2	85±3	72±3	63±2
1e	Me	Me	Me	3-chloropyrrol-2-yl	99±2	100±1	103±2	105±2	103±2	101±3	70±2	59±3
1f	Me	Et	Me	3-chloropyrrol-2-yl	101±2	100±2	110±2	101±2	101±2	100±2	76±3	75±2
1g	*n*Bu	Me	Me	3-chloropyrrol-2-yl	88±2	80±3	98±3	78±2	92±2	90±2	80±3	60±3
1h	H	H	H	3-chloropyrrol-2-yl	106±3	105±3	115±3	102±3	101±2	105±2	88±3	79±3
1i	Me	H	H	3-chloropyrrol-2-yl	103±2	104±2	102±2	106±2	96±2	100±3	85±3	76±2
1j	H	H	Me	3-chlorothiophen-2-yl	107±5	108±3	80±3	80±2	97±3	98±2	62±3	45±3
1k	Me	H	Me	3-chlorothiophen-2-yl	105±2	100±1	101±2	100±2	101±3	101±2	87±3	86±2
1l	Me	H	Me	2-chlorophenyl	108±2	104±2	102±2	98±2	102±2	103±3	90±2	75±3
1m	Me	H	Me	3-chlorophenyl	110±3	103±2	103±3	105±2	99±3	102±3	88±2	80±2
1n	Me	H	Me	3-chloro-1-mesyl-pyrrol-2-yl	116±5	109±6	120±6	99±6	100±3	101±2	93±6	100±2
21	Me	H	Me	H	95±2	100±2	88±3	90±3	98±3	103±2	90±2	79±3
paclitaxel					58±2	56±3	78±2	57±3	92±2	94±3	66±2	45±3

a The concentration of the tested compound is 20 µM; the concentration of paclitaxel is 50 nM.

b The cell viability are expressed in % of control as mean ± SD of three independent experiments.

### Compound 1a Induces Mitochondrial Death Pathway and Caspase-dependent Apoptosis

**Figure 3:**
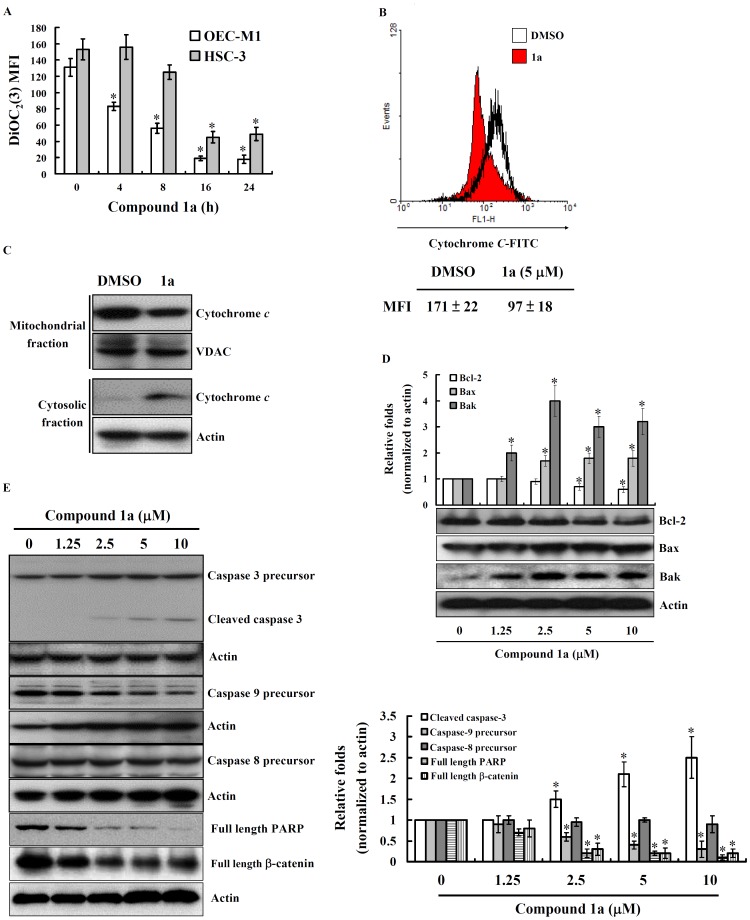
Compound 1a induces mitochondrial death pathway and caspase-dependent apoptosis. (**A**) Cells were stimulated with compound **1a** (5 µM) or vehicle (0.1% DMSO) for the time indicated, followed by DiOC_2_(3) staining. The mitochondrial membrane potential was analyzed by flow cytometry. The results were expressed as mean ± SD of mean fluorescence intensity (MFI); n = 3. (**B**) OEC-M1 cells were stimulated with compound **1a** (5 µM) or vehicle for 16 h, the cytochrome *c* in mitochondria was measured by flow cytometry. The results were expressed as mean ± SD of MFI; n = 3. (**C**) The cytochrome *c* in mitochondrial fraction and cytosolic fraction were measured by western blotting. Voltage-dependent anion channel (VDAC) and actin were used as a marker protein for mitochondria and cytosol respectively. One of three repeated experiments was shown. (**D**) OEC-M1 cells were treated with various concentration of compound **1a** as indicated or vehicle for 24 h. The expression levels of Bcl-2, Bax and Bak were measured by western blotting. (**E**) OEC-M1 cells were treated with various concentration of compound **1a** as indicated or vehicle for 24 h. The expression levels of caspase 3 precursor, cleaved caspase 3, caspase 9 precursor, caspase 8 precursor, PARP and β-catenin were measured by western blotting. In (**D**) and (**E**), the results are representative of those obtained in three different experiments and the histogram shows the quantification expressed as the mean ± SD. *indicates a significant difference at the level of *p*<0.05 compared to control group.

Mitochondria play an important role in cell death by changing its outer and inner membrane permeability and thus leading to cytochrome *c* release and caspase activation [21]. To explore whether compound **1a** induced apoptosis via the mitochondrial signaling pathway, the mitochondrial membrane potential alteration was determined using a mitochondria-specific fluorescence dye, DiOC_2_(3). OEC-M1 and HSC-3 cells treated with compound **1a** were found to demonstrate a loss of fluorescence intensity with time (Fig. 3A), indicating that compound **1a** induces a loss of mitochondrial membrane potential. Activation of the mitochondrial death pathway can also be identified by the release of mitochondrial cytochrome *c*. Cytosolic cytochrome *c* was detected by varying the exposure of OEC-M1 cells to compound **1a** and the level of cytochrome *c* that remained in the mitochondria was observed to decrease concomitantly (Fig. 3B). The release of cytochrome *c* from mitochondria to cytosol was confirmed by western blotting (Fig. 3C). To get insights into the mechanism of compound **1a**-induced mitochondrial mediated cell death, we examined the expression of Bcl-2 family proteins including Bcl-2, Bax and Bcl-2 homologous antagonist killer (Bak). The amount of anti-apoptotic protein, Bcl-2, was observed to decrease after treatment with compound **1a** (Fig. 3D). In contrast, the expression of pro-apoptotic protein, Bax and Bak, increased in the presence of compound **1a** (Fig. 3D). As shown in Fig. 3E, compound **1a** induced a decrease of caspase-9 precursor and an increase of cleaved caspase-3 in a concentration-dependent manner, but compound **1a** did not induce caspase-8 activation. The activation of caspase-3 was further confirmed by detecting the degradation of PARP and β-catenin which were cleaved by the active caspase-3 during apoptosis (Fig. 3E).

### Compound 1a-induced Apoptosis is Dependent on Oxidative Stress

**Figure 4:**
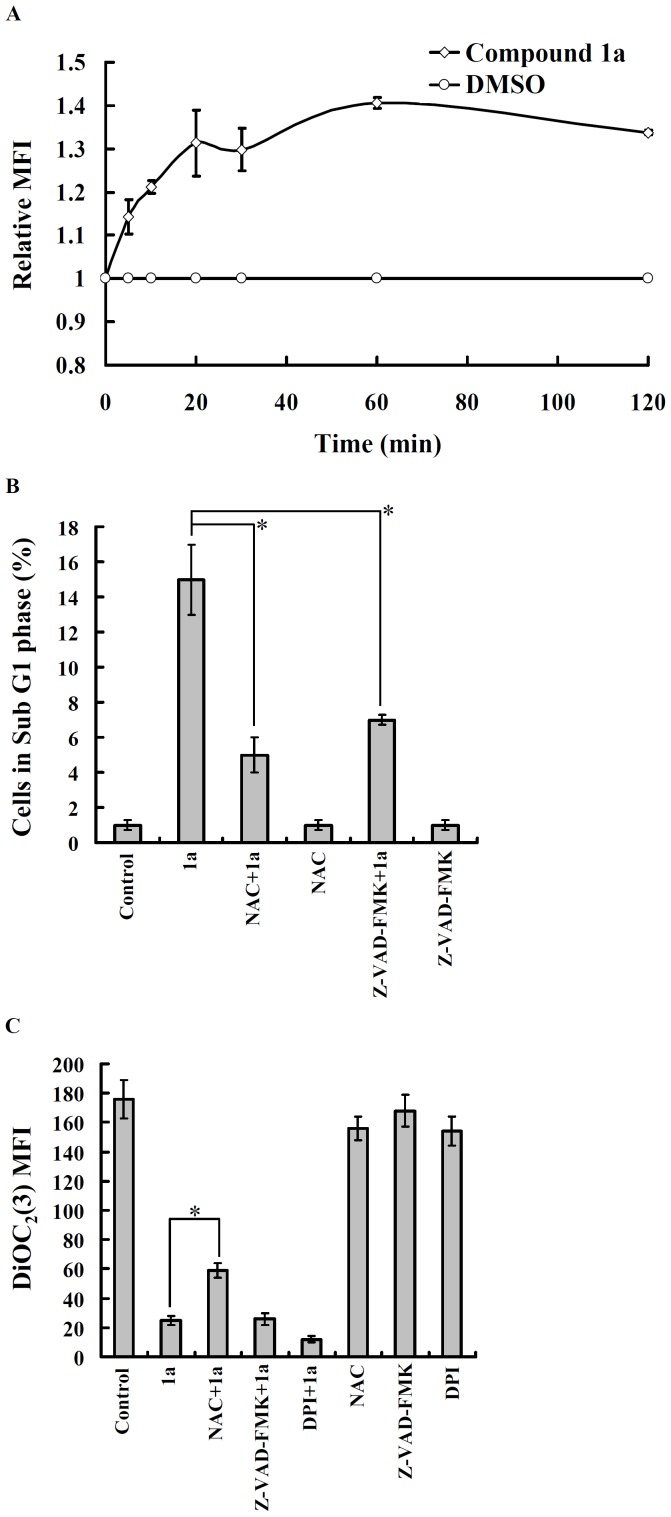
Compound 1a-induced apoptosis is dependent on oxidative stress. (**A**) OEC-M1 cells were incubated with 2′,7′-dichlorofluorescein diacetate (2 µM) for 30 min, followed by compound **1a** (5 µM) or vehicle (0.1% DMSO) treatment for the time indicated. The relative mean fluorescence intensity (MFI) of 2′,7′-dichlorofluorescein was detected at an excitation wavelength of 485 nm and an emission wavelength of 530 nm with a fluorometer; n = 3. (**B**) OEC-M1 cells were incubated with *N*-acetyl-cysteine (NAC) (10 mM) or Z-VAD-FMK (50 µM) for 30 min, followed by compound **1a** (5 µM) or vehicle treatment for 24 h. The cell cycle distribution was determined by flow cytometry after PI-staining of nuclei. The data were expressed as mean ± SD; n = 3. (**C**) OEC-M1 cells were incubated with NAC (10 mM), Z-VAD-FMK (50 µM), or DPI (25 µM) for 30 min, followed by compound **1a** (5 µM) or vehicle treatment for 16 h. The mitochondrial membrane potential was analyzed by flow cytometry using DiOC_2_(3) staining. The data are expressed as mean ± SD of MFI; n = 3. *indicates a significant difference at the level of *p*<0.05.

To investigate the possible involvement of ROS in compound **1a**-induced apoptosis, intracellular ROS stimulated by compound **1a** was measured by detecting the fluorescence intensity of 2′,7′-dichlorofluorescein, the oxidation product of 2′,7′-dichlorofluorescein diacetate. OEC-M1 cells stimulated with compound **1a** exhibited a rapid increase in the fluorescent product 2′,7′-dichlorofluorescein which resulted in the relative fluorescence intensity peaking at 60 min (Fig. 4A). We next investigated whether ROS plays a role in compound **1a**-induced apoptosis. We found that antioxidant *N*-acetylcysteine (NAC) decreased compound **1a**-mediated sub-G1 phase increase in OEC-M1 cells (Fig. 4B). NAC, but not pan caspase inhibitor Z-VAD-FMK and NADPH oxidase inhibitor diphenyleneiodonium (DPI), partially prevented the loss of mitochondrial membrane potential induced by compound **1a** (Fig. 4C).

### Compound 1a-induced Apoptosis and DNA Damage through Cytochrome P450

**Figure 5:**
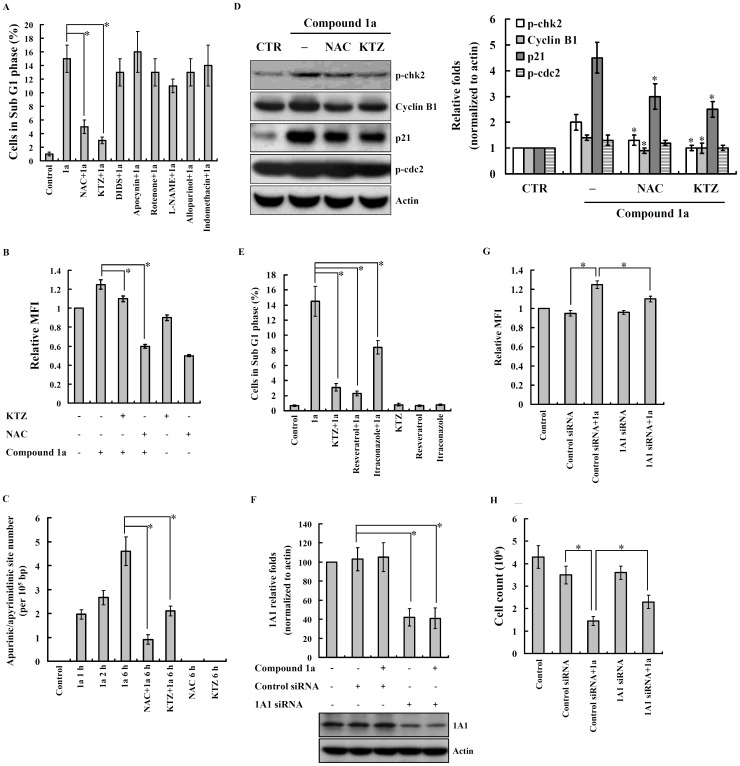
Compound 1a-induced apoptosis and DNA damage through cytochrome P450. (**A**) OEC-M1 cells were incubated with NAC (10 mM), ketoconazole (KTZ, 50 µµM), diisothiocyanatostilbene-disulfonic acid (DIDS, 10 µM), apocynin (0.3 µM), rotenone (1 µM), L-NAME (500 µM), allopurinol (100 µM), indomethacin (50 µM) for 30 min, followed by compound **1a** (5 µM) or vehicle (0.1% DMSO) treatment for 24 h. The cell cycle distribution was determined by flow cytometry after PI-staining of nuclei. (**B**) OEC-M1 cells were incubated with KTZ (50 µM) and NAC (10 mM) in the presence of 2′,7′-dichlorofluorescein diacetate (2 µM) for 30 min, followed by compound **1a** (5 µM) or vehicle (0.1% DMSO) treatment for 30 min, and then the ROS production was measured. (**C**) OEC-M1 cells were incubated with NAC (10 mM) or ketoconazole (KTZ, 50 µM) for 30 min, followed by compound **1a** (5 µM) or vehicle treatment for the time as indicated. AP sites of DNA lesions were analyzed by a DNA damage quantification kit. (**D**) OEC-M1 cells were treated with NAC (10 mM) or ketoconazole (KTZ, 50 µM) for 30 min, followed by compound **1a** (5 µM) or vehicle (0.1% DMSO) treatment for 2 or 16 h. The expression levels of p-chk2 (2 h), cyclin B1, p21, and p-cdc2 were measured by western blotting. (**E**) OEC-M1 cells were incubated with resveratrol (100 µM) or itraconazole (10 µM) for 30 min, followed by compound **1a** (5 µM) or vehicle treatment for 24 h. The cell cycle distribution was determined by flow cytometry after PI-staining of nuclei. (**F**) Untreated OEC-M1 cells, cytochrome P450 1A1 siRNA transfected OEC-M1 cells, and control siRNA transfected OEC-M1 cells were incubated with compound **1a** (5 µM) or vehicle for 24 h and then the cytochrome P450 1A1 expression were measured by western blotting. (**G**) Untreated OEC-M1 cells, cytochrome P450 1A1 siRNA transfected OEC-M1 cells, and control siRNA transfected OEC-M1 cells were incubated with compound **1a** (5 µM) or vehicle for 30 min and then the ROS production was measured. (**H**) Untreated OEC-M1 cells, cytochrome P450 1A1 siRNA transfected OEC-M1 cells, and control siRNA transfected OEC-M1 cells were incubated with compound **1a** (5 µM) or vehicle for 24 h. The cell number was determined by trypan blue staining. In (**A**), (**B**), (**C**), (**E**), (**G**) and (**H**), the data are expressed as mean ± SD of three independent experiments, while, in (**D**) and (**F**), the results are representative of those obtained in three different experiments and the histogram shows the quantification expressed as the mean ± SD. *indicates a significant difference at the level of *p*<0.05.

We further investigated which intracellular ROS-generating enzymes were involved in compound **1a**-mediated apoptosis. OEC-M1 cells were stimulated with compound **1a** in the presence or absence of NAC (scavenger of ROS regardless of the source of production), ketoconazole (cytochrome P450 inhibitor), apocynin (NADPH oxidase inhibitor), DIDS (mitochondrial inner membrane anion channel inhibitor), rotenone (mitochondrial complex I inhibitor), L-NAME (iNOS inhibitor), allopurinol (xanthine oxidase inhibitor), and indomethacin (cyclooxygenase inhibitor) for 30 min, and then the cell cycle distribution was determined. We found that NAC and ketoconazole not only decreased the cells in sub-G1 phase induced by compound **1a** (Fig. 5A), but also reduced the compound **1a**-induced increase in ROS generation (Fig. 5B). Since compound **1a** induces ROS generation, this prompted us to investigate whether compound **1a** damages DNA. Using the apurinic/apyrimidinic (AP) sites formation assay, we found that compound **1a** causes DNA damage and the damage is decreased by NAC and ketoconazole (Fig. 5C). NAC and ketoconazole were also found to modulate G2/M phase regulatory proteins by reducing phosphorylation level of Chk2 and by reducing the expression levels of cyclin B1 and p21 in compound **1a**-stimulated OEC-M1 cells (Fig. 5D). In addition, ketoconazole inhibited both cytochrome P450 1A1 and 3A4, and we further found that resveratrol (cytochrome P450 1A1 inhibitor) decreased sub G1 increase induced by compound **1a** more significantly than itraconazole (cytochrome P450 3A4 inhibitor) (Fig. 5E). Since resveratrol is not a specific inhibitor to cytochrome P450 1A1, the role of cytochrome P450 1A1 on compound **1a**-mediated effects was confirmed by siRNA. Knocking down the expression of cytochrome P450 1A1 by siRNA (Fig. 5F) was shown to decrease compound **1a**-mediated ROS production (Fig. 5G) and cytotoxicity (Fig. 5H).

### Anti-tumor Activity of Compound 1a *in vivo*

**Figure 6:**
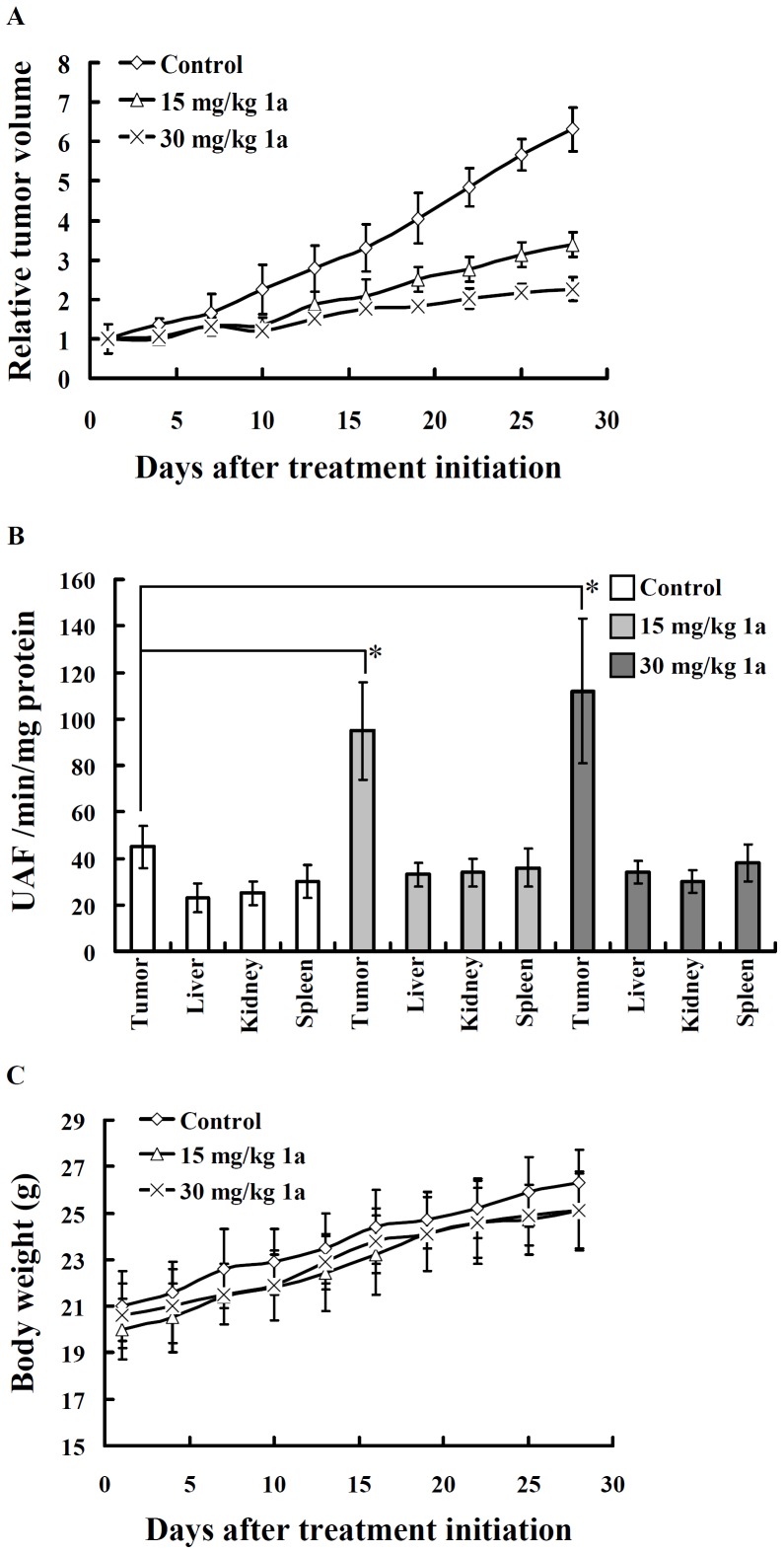
Inhibition of human oral cancer xenografts growth in vivo by compound 1a. (**A**) OEC-M1 tumor-bearing mice were administered s.c. with vehicle control (◊), 15 mg/kg (△), or 30 mg/kg (×) compound **1a** on days 0–4 for 5 days. The figure showed the relative tumor volume of control and compound **1a**-treated groups. (**B**) Caspase 3 activity of tissues from OEC-M1 tumor-bearing mice (on day 28 after inoculation). (**C**) The changes of body weight of the tumor-bearing mice. Body weight was measured for each animal every three days starting from the day of the initial compound **1a** treatment. All results are given as means ± SD; n = 6 for each group.*indicates a significant difference at the level of *p*<0.05.

Results obtained show that compound **1a** inhibited the growth of tumor (Fig. 6A). The tumor tissues from compound **1a** treated mice displayed an increase in caspase 3 activity compared to that from vehicle treated mice, whereas the caspase-3 activity in the liver, kidney, and spleen of compound **1a** treated or vehicle treated mice was not increased (Fig. 6B). Additionally the changes in body weight of the compound **1a**-treated mice were similar to that of the vehicle-treated mice (Fig. 6C).

## Discussion

In our previous study [13], the most potent gymnoconjugatin derivatives against human lung cancer A549 cells was compound **1g** (IC_50_ values of 0.01 µM); however, in the present study, compound **1g** did not showed potent cytotoxicity against OSCC. Compound **1a** was identified as the most potent compound against OSCC. The loss of activity against OSCC in compounds **1j**–**1n**, where other chloro-substituted aromatic rings were present instead of 3-chloropyrrole, indicated that the later group played an important role in effecting cytotoxicity. In addition, the lack of cytotoxicity in compounds **1h** and **1i** also illustrated the importance of a methyl group at the R^3^ position. Similar to our previous study, compounds **1j**-**1n**, **1h** and **1i** did not show cytotoxicity against A549 cells [13]. The similarity between compounds **1a** and **1b** suggests that they might show similar cytotoxicity. Compounds **1a** and **1b** showed cytotoxicity against A549 cells with IC_50_ values of 0.6 µM and 1.2 µM respectively [13]; interestingly, compound **1b** lacks cytotoxicity against OEC-M1 and HSC-3 cells. These results indicated that having a hydrogen at the R^1^ position is important for its cytotoxicity against OEC-M1 and HSC-3 cells. The relation between chemical structure and cell-type specificity needs to be further investigation. Four OSCC cell lines were used in this study, OEC-M1 cells were an indigenous oral cancer cell line in Taiwan with a p53 point mutation at codon 173 (V to L) and were more sensitive to radiation, but more resistant to chemotherapy [22], [23]. The SCC-4 cell line expresses and accumulates p53 with a missense mutation/point mutation in position 151, whereas HSC-3 lacks p53 [24]. SAS cells have a point mutation in one p53 allele, but it has been shown to possess an ability to induce p53-dependent signal transduction [25]. The viability of OEC-M1 cells, SCC-4 cells, and HSC-3 cells were all reduced by compound **1a**; however, the killing effect of compound **1a** on these cell lines might not be mediated by p53. In contrast to OEC-M1 cells, SAS cells have a high level expression of EGFR [26], and is resistant to TRAIL-induced apoptosis [27]. In this study, SAS cells were shown to be resistant to compound **1a** treatment and we have yet to find the cause for this resistance.

Intracellular ROS accumulation is involved in the induction of apoptosis by various cytotoxic agents [28]. In the present study, ROS was demonstrated to be pro-apoptotic in compound **1a**-treated oral cancer OEC-M1 cells by showing a protective effect of antioxidants on DNA damage, mitochondrial membrane potential loss, and apoptosis. In contrast, ROS played an anti-apoptotic and protective role in compound **1a**-treated lung cancer A549 cells [13]. The role of oxidative stress in apoptosis is controversial and could be stimulus and cell type dependent [29], [30]. ROS are mainly produced by mitochondria and NADPH oxidase; however, inhibitors of mitochondria and NADPH oxidase did not reduce compound **1a**-induced apoptosis. Cytochrome P450 superfamily is a diverse group of enzymes that catalyzes the oxidation of various substances and is involved in drug metabolism (either directly or by facilitated excretion from the body) and bioactivation (transforming into their active compounds) [31]. For example, cytochrome P450 1A1 is involved in the metabolic activation of aromatic hydrocarbons, such as benzo(a)pyrene, by transforming it to carcinogen BP-7,8-dihydrodiol-9,10-epoxide [32]. ROS generated by cytochrome P450 1A1 contributes to carcinogenesis [33], [34], hypertension [35], and tissue damage [36]. In the present study we found that cytochrome P450 1A1 played a pro-apoptotic role in compound **1a**-treated OEC-M1 cells. The pro-apoptotic effect of cytochrome P450 might proceed via the transformation of compound **1a** to a more toxic compound or through the production of ROS. It has been suggested that cytochrome P450 1A1 promotes DNA damage and G2/M arrest in benzo[a]pyrene-stimulated breast carcinoma MCF-7 cells [37]. In contrast, cytochrome P450 1A1 is able to detoxify toxins, for examples, it plays a protective role in benzo[a]pyrene-induced oral cancer in mice [38] and in arsenic toxicity in rat liver cells by reducing apoptosis [39]. Cytochrome P450 1A1 polymorphism on exon 7 has also be shown to be capable of modifying the susceptibility to oral cancer among Asians [40].

The anti-cancer activity of the p53 protein has been demonstrated by its ability to induce apoptosis in response to a variety of cellular stresses including anti-cancer drugs. P53 promotes apoptosis through down-regulating Bcl-2 protein expression and up-regulating BAX protein expression at transcriptional level [41], [42]. P53 also promotes mitochondrial apoptosis by activating BAK protein [43]. Our data revealed that the protein levels of p53 and p21 were increased by compound **1a**. These suggest that p53 activation may be involved in compound **1a**-mediated apoptosis which in turn regulates Bcl-2 family protein expression. However, in the present study we should not rule out the possibility for compound 1**a**-induced increase in p21 expression and the changes in the Bcl-2 family protein expression in OEC-M1 cells would then occur in a p53-independent manner.

**Figure 7:**
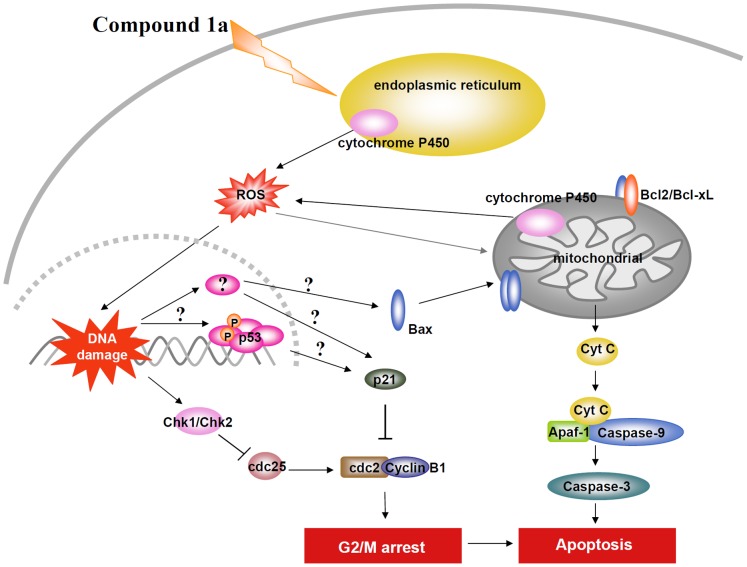
The proposed mechanism for compound 1a-mediated apoptosis in human oral squamous cell carcinoma cells.

In the present study we clearly demonstrated that compound **1a** is efficacious against OSCC cells both in cell culture and animal models. Mechanistic studies suggested that compound **1a** causes DNA damage by enhancing intracellular ROS level which leads to cell cycle arrest and apoptotic cell death. The latter phenomenon is also partially dependent on cytochrome P450 1A1 (Fig. 7). Considering the limited therapeutic options available against OSCC, results from the present study provides a molecular rationale for the translation of compound **1a** into a potential therapeutic against OSCC.
